# A New Measure of Imagination Ability: Anatomical Brain Imaging Correlates

**DOI:** 10.3389/fpsyg.2016.00496

**Published:** 2016-04-18

**Authors:** Rex E. Jung, Ranee A. Flores, Dan Hunter

**Affiliations:** ^1^Department of Psychology, University of New MexicoAlbuquerque, NM, USA; ^2^Department of Neurosurgery, University of New MexicoAlbuquerque, NM, USA; ^3^Hunter Higgs, LLCBoston, MA, USA

**Keywords:** imagination, creativity, brain volume measurements, neuroimaging (anatomic and functional), nucleus accumbens (NAcc), lingual gyrus

## Abstract

Imagination involves episodic memory retrieval, visualization, mental simulation, spatial navigation, and future thinking, making it a complex cognitive construct. Prior studies of imagination have attempted to study various elements of imagination (e.g., visualization), but none have attempted to capture the entirety of imagination ability in a single instrument. Here we describe the Hunter Imagination Questionnaire (HIQ), an instrument designed to assess imagination over an extended period of time, in a naturalistic manner. We hypothesized that the HIQ would be related to measures of creative achievement and to a network of brain regions previously identified to be important to imagination/creative abilities. Eighty subjects were administered the HIQ in an online format; all subjects were administered a broad battery of tests including measures of intelligence, personality, and aptitude, as well as structural Magnetic Resonance Imaging (sMRI). Responses of the HIQ were found to be normally distributed, and exploratory factor analysis yielded four factors. Internal consistency of the HIQ ranged from 0.76 to 0.79, and two factors (“Implementation” and “Learning”) were significantly related to measures of Creative Achievement (Scientific—*r* = 0.26 and Writing—*r* = 0.31, respectively), suggesting concurrent validity. We found that the HIQ and its factors were related to a broad network of brain volumes including increased bilateral hippocampi, lingual gyrus, and caudal/rostral middle frontal lobe, and decreased volumes within the nucleus accumbens and regions within the default mode network (e.g., precuneus, posterior cingulate, transverse temporal lobe). The HIQ was found to be a reliable and valid measure of imagination in a cohort of normal human subjects, and was related to brain volumes previously identified as central to imagination including episodic memory retrieval (e.g., hippocampus). We also identified compelling evidence suggesting imagination ability linked to decreased volumes involving the nucleus accumbens and regions within the default mode network. Future research will be important to assess the stability of this instrument in different populations, as well as the complex interaction between imagination and creativity in the human brain.

## Introduction

The ability to imagine oneself carrying out activities in the future is an important aspect of both creative cognition and creative achievement. There is a fairly long history of linking imagination to creativity, with early researchers seeing imagination as a subset of the broader construct of creative cognition, especially in developmental disorders (Craig and Baron-Cohen, 1999). More recently, imagination has been conceptualized as a critical mediating linkage between acquired knowledge and creative insight, constraining the possible solutions through mental simulations or “incubation” (Duch, 2007; Helie and Sun, 2009). This “imaginative” aspect of creativity is not assessed by current measures of imagination (proper), most of which focus on visualization, or imagery (Zhang et al., 2012), or are quite similar to standard measures of divergent thinking (Jankowska and Karwowski, 2015).

An operational definition of imagination likely involves aspects of episodic memory retrieval, visualization, simulation, spatial navigation, and future thinking, but these are pieces of a bigger puzzle, comprising various stages of the creative process, from preparation, through incubation, illumination, and verification (Poincare, 1913). At what level of resolution should we parse this important human attribute? While imagination is certainly dependent upon fundamental cognitive processes including attention, semantic memory retrieval, working memory (the list goes on and on), we aim to define and measure this construct *in toto* in spite of the temptation to fragment it into less interesting (albeit scientifically submissive) parts. Thus, the understanding of imagination, as a critical component of creative cognition, is the aim of this study. As part of this understanding, we endeavor to describe, for the first time, anatomical correlates of imagination ability in normal human participants.

Classic studies of imagery, a component of “imagination” in patients suffering hippocampal damage, ask questions such as “Imagine you are lying on a white sandy beach in a beautiful tropical bay,” and ask them to describe what they see (Hassabis et al., 2007). While these studies get at imagination through visualization, they do not ask participants to generate ideas related to their own lives or work (i.e., episodic memory retrieval), nor do they ask them to think about themselves in the future (i.e., future thinking), cognitive processes hypothesized to be important to creativity (Jung et al., 2015; Beaty et al., 2016a; Crespi et al., 2016). Second, these studies of imagery do not allow ideas to incubate over time, but are often “snapshot” representations of impressions captured in the moment. While comprehensive imagination capacity is anticipated to be difficult to capture with either brain or behavioral measures, we adopt Simonton's “test” of a “most desirable” measure: applicable to different domains and ability levels, and not suffering from excessive granularity (Simonton, 2012).

Imagination is a large cognitive construct. However, if imagination involves fundamentally interwoven cognitive processes including memory, visualization, spatial navigation, and episodic future thinking, these processes should involve concomitant neural structures associated with their behavioral manifestation. For example, the hippocampus has been well associated with episodic memory formation, extending from the unfortunate case of HM, who underwent bilateral resection of his hippocampi as a cure for intractable epilepsy, rendering him unable to form new memories (Scoville and Milner, 1957). Studies with rats have also demonstrated location-specific firing within the hippocampus, with damage to this structure resulting in disrupted spatial navigation ability (O'keefe and Nadel, 1978). When participants with acquired hippocampal damage are asked to imagine themselves in various scenes, they do so with great effort, and with lower spatial contiguity and coherence (Hassabis et al., 2007). Outside of the medial temporal lobe *per se*, a broader network of regions has been implicated in imagination. This network includes a “core” within the hippocampus, parahippocampus, posterior cingulate and posterior parietal cortices, and “secondary” and “infrequent” involvement of medial/lateral prefrontal and lateral temporal cortices, associated with concepts of self/other and mental time travel (Nyberg et al., 2010).

Based on our review above, we define imagination as drawing upon previous experiences to engage in mental simulation, in order to achieve future goals. We describe a measure of imagination, the Hunter Imagination Questionnaire (HIQ), designed to (1) capture aspects of memory retrieval, visualization, simulation, spatial navigation, and episodic future thinking, (2) capture imagination activities over an extended period of time, and (3) ask participants to envision future goals and achievements. We hypothesized that, if participants engaged in such imagination activities, then associated brain networks, identified previously within the neuroscientific literature, would be involved in their responses, particularly those at the core of imagination (e.g., medial temporal) as well as those involved in thinking about oneself vs. others, and mental time travel (e.g., medial frontal, lateral temporal).

## Methods

This study was conducted according to the principles expressed in the Declaration of Helsinki, and was approved by the Institutional Review Board of the University of New Mexico (IRB#11-531). All participants provided written informed consent prior to collection of any experimental samples and subsequent data analysis. Eighty participants (29 males; 51 females) between the ages of 16 and 35 (Mean = 22.5; SD = 4.3) were recruited from the University of New Mexico and surrounding community. Participants were screened by questionnaire to exclude major neurological injury or disease (e.g., traumatic brain injury, epilepsy) and psychiatric disorder (e.g., major depression, attention deficit disorder). All participants underwent a magnetic resonance imaging (MRI) session, including measures of brain structure, diffusion tensor imaging, and functional measures of the default mode network (DMN).

### Behavioral measures

Participants were administered behavioral measures including the HIQ. All participants had previously completed a battery of measures including tests of intelligence (Wechsler Abbreviated Scale of Intelligence—WASI), personality (Big 5 Aspect Scale—BFAS), and aptitude (Paper Folding, Vocabulary, Foresight), and received $100 compensation for their time. The WASI is a standardized measure of intelligence, used in both clinical and educational testing to derive an intelligence quotient (IQ) from ages 6 to 90 (Wechsler, 1999). It is comprised of subtests including Vocabulary, Similarities (e.g., how are green and red alike), Block Design, and Matrix Reasoning; we administered all subtests but Vocabulary, which was obtained from an aptitude measure described below. The BFAS is self-report measure, consisting of 100 items, of non-clinical personality domains including Neuroticism, Extroversion, Openness, Agreeableness, and Conscientiousness (DeYoung et al., 2007). The facet of Openness has been well associated with creative cognition (McCrae and Ingraham, 1987; Miller and Tal, 2007; Kaufman et al., 2014) and linked to brain measures within the default mode network (Sampaio et al., 2014; Beaty et al., 2016b). Paper Folding, Vocabulary, and Foresight are measures of aptitude from the Johnson O'Connor battery of tests. Paper Folding measures the ability to mentally manipulate paper forms having holes punched out of them in different patterns; Vocabulary measures single word knowledge, in a multiple choice format, with a range of words presented from easy (e.g., plump) to quite difficult (e.g., mephitic); Foresight measures the ability to generate as many ideas about visual designs as possible in 45 s. These measures, and their anatomical correlates, have been reported by our group previously (Jung et al., 2014, 2015). We were particularly interested in the relationship between the HIQ and the Creative Achievement Questionnaire (CAQ). The CAQ is a reliable and valid measure of creative achievement across 10 domains including visual arts, music, creative writing, dance, drama, architecture, humor, scientific discovery, invention, and culinary arts (Carson et al., 2005).

### Procedure

Participants were drawn from a larger pool of participants who were being studied to determine individual differences in creative cognition and aptitude reported on previously (Jung et al., 2014, 2015). As part of this larger study, all participants underwent a 4-h battery of measures including tests of intelligence, personality, and aptitude. All of these measures were administered in a laboratory setting, with research assistants utilized to administer tests requiring individual administration (e.g., WASI, Vocabulary, Paper Folding, Foresight, CAQ). Participants underwent a separate neuroimaging session, usually within 1 month of individual testing, where they underwent anatomical Magnetic Resonance Imaging (aMRI), Diffusion Tensor Imaging (DTI), and an echoplanar session designed to elicit the Default Mode Network of brain functioning. This imaging session took no longer than ½ h to complete.

For the current study, all participants from the larger sample were asked to participate in an online questionnaire where they were asked several questions about their imagination activity. Eighty of 246 participants agreed to participate in this subsequent questionnaire, and were sent instructions regarding how to access an online portal where their responses were recorded (REDCap). Participants were paid $50 for their time.

Participants were given instructions to complete Session 1 of the HIQ, and to submit their responses to the cue (below) in the REDCap system. Participants were instructed that they should take no longer than 8 min to complete Session 1.

#### Session 1 cue

What would you like to do, make, create, or achieve in the next few months? You may include both feasible and fantastical ideas. Write as much as you need to be able to remember your ideas. Begin each new idea on a new line. Try to generate 3–5 (or more) ideas in 8 min. When you are done hit the “submit” button at the end of the page.

Following 3 days, participants were sent an email with a link to complete Session 2 of the HIQ. Participants were given instructions to complete Session 2 of the HIQ, and to submit their responses to the REDCap system. If they did not respond within 1 week, they were sent one email reminder to complete Session 2.

#### Session 2 cue

Visualize a scene in your mind invoking your senses. The scene may be realistic or fantastical; landscape, or interior. Write as much detail as you need to remember the scene. Try to visualize 3–5 (or more) scenes in 8 min. When you are done hit the “submit” button at the end of the page.

Following 3 days, participants were sent an email with a link to complete Session 3 of the HIQ. Participants were given instructions to complete Session 3 of the HIQ, and to submit their responses to the REDCap system. If they did not respond within 1 week, they were sent one email reminder to complete Session 3.

#### Session 3 cue

Imagine something you would like to discover or invent or change. It could be real or imaginary. Begin each new idea on a new line. Try to generate 3–5 (or more) scenes in 8 min. When you are done hit the “submit” button at the end of the page.

Following 3 days, participants were sent an email with a link to complete Session 4 of the HIQ. Participants were given instructions to complete Session 4 of the HIQ, and to submit their responses to the REDCap system. If they did not respond within 1 week, they were sent one email reminder to complete Session 4.

#### Session 4 cue

What would you like to do, make, create, or achieve in the next few months? You may include both feasible and fantastical ideas. Write as much as you need to be able to remember your ideas. Begin each new idea on a new line. Try to generate 3–5 (or more) ideas in 8 min. When you are done hit the “submit” button at the end of the page.

Following 3 days, participants were sent an email with a link to complete the Review of Ideas of the HIQ. They were instructed to review all of their ideas and notes from the last four sessions and to consider which appealed to them the most, which ideas they will implement, and which they are most likely to forget or not implement. They were instructed to select (type) three of their best ideas. This was followed by a set of questions ranked on a scale from 1 (low) to 10 (high).

1. How passionate or engaged are you with the ideas you generated? (1 = Disengaged/Bored; 10 = Passionate/Engaged).
2. Have you taken steps to implement any of your ideas? (1 = No Steps Taken; 10 = Idea Completed).
3. How likely are you to implement or continue implementing your ideas in the days and weeks to come? (1 = Unlikely; 10 = Very Likely).
4. How difficult was the first session? (1 = Not Difficult at All; 10 = Very Difficult).
5. Did the process become easier or more difficult as you repeated the assessment? (1 = Easier; 10 = More Difficult).
6. Please estimate how much time you devoted to thinking about your ideas between sessions. (1 = No Time; 10 = A Lot of Time).
7. Are you satisfied with the number of ideas you generated? (1 = Not Satisfied; 10 = Completely Satisfied).
8. Did the assessment process help you learn about your own thinking? (1 = Not At All; 10 = Quite A Bit).
9. How would you rate your experience of the assessment? (1 = Very Bad; 10 = Very Good).
10. Please provide an overall assessment of your ideas on a scale of 1–10 (1 = Very Bad; 10 = Very Good).

**Figure 1:**
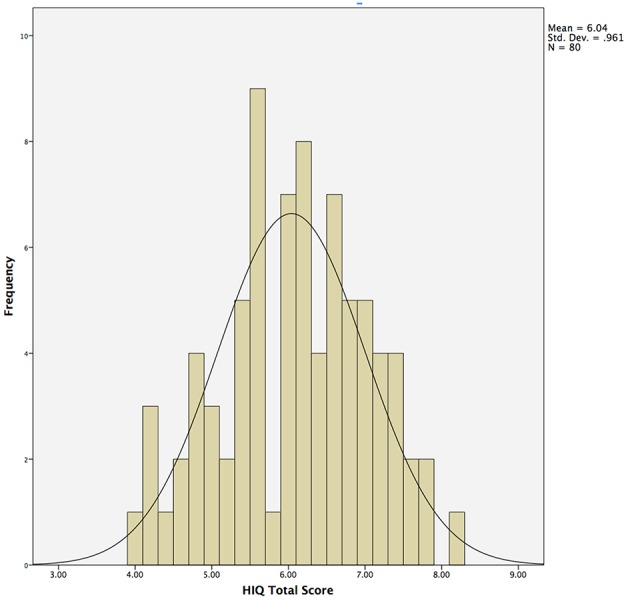
**Histogram demonstrating normal distribution of Hunter Imagination Questionnaire (HIQ) total scores across the entire sample (*N* = 80)**.

Questions from Session 1 and 4 were never presented sequentially, and questions were presented to participants in pseudorandom order to control for order effects. All questions were answered on a Likert scale ranging from 1 (not at all, unlikely, etc.) to 10 (high, quite a bit, likely, etc.). The HIQ Total Score was obtained by summing scores obtained on items 1 through 10, and dividing by 10 (Figure 1).

### Neuroimaging

Anatomical imaging was obtained using a 3 Tesla Siemens scanner using a 32-channel head coil. We obtained a T1 5 echo sagittal MPRAGE sequence (TE = 16.4; 3.5; 5.36; 7.22; 9.08 ms; TR = 2530 ms; voxel size = 1.0 × 1.0 × 1.0 mm3; slices = 192; acquisition time = 6:03). Methods for cortical reconstruction and volumetric segmentation were performed with the FreeSurfer image analysis suite (http://surfer.nmr.mgh.harvard.edu/) and are described in detail elsewhere (Fischl et al., 2002, 2004; Han and Fischl, 2007). Thickness measurements were obtained by reconstructing representations of the Gray Matter/White Matter boundary and the pial surface and then calculating the distance between those surfaces at each point across the cortical mantle (Dale et al., 1999). The results of the automatic segmentations were quality controlled and any errors were manually corrected. Volume measures are a combination of thickness (a one-dimensional measure) and area (a two dimensional measure) across 33 measures per hemisphere (i.e., 66 across the surface of the brain) as well as seven subcortical volumes per hemisphere (i.e., 14 across the brain) including bilateral caudate, putamen, globus pallidus, nucleus accumbens, thalamus, amygdala, and hippocampus (Fischl et al., 2002).

### Analysis

We used Shapiro–Wilk to test normality of the distribution of the HIQ. Student's t was used to test for differences between males and females on major variables of interest, including all scores on the HIQ. Exploratory factor analysis, with Principal Axis Factoring, Varimax rotation, and Kaiser Normalization was used to characterize the structure of items on the HIQ. Cronbach's alpha was used to determine internal consistency of items within each factor. Partial correlation, controlling for sex, was used to determine the relationship between total scores and factor scores of the HIQ and total scores and item scores on the CAQ. Finally, linear regression, controlling for age, sex, Full Scale Intelligence Quotient, and Total Supratentorial Volume was used to determine the relationship between the HIQ factor scores and brain volume measures. There are 80 (66 cortical and 14 subcortical) volumes obtained across the brain for each participant. Given that five in 100 Type I errors are considered to be generally acceptable in research designs, we would expect roughly four regions of 80 to be related to our measures by chance. We have adjusted our significance levels to *P* < 0.005 to account for such possible chance relationships as in our previous research (Jung et al., 2015). While this does not fully account for Type I error, we believe that it reasonably balances the risk of both Type I and Type II error in this exploratory experiment.

## Results

### Normality

Responses on the HIQ were normally distributed (Shapiro–Wilk = 0.987), with a mean of 6.0 and standard deviation of 1.0 (Table 1; Figure 1). Males (*N* = 29) did not differ significantly from females (*N* = 51) in overall scores, although males tended to score slightly higher overall (Male Mean = 54.8; Female Mean = 52.8), largely driven by significant differences in satisfaction (Male Mean = 6.7; Female Mean = 5.5; *t* = 2.0, *p* = 0.05). Means and standard deviations for all behavioral measures are presented in Table 1.

**Table 1:** **Descriptive statistics for participants on behavioral measures**.

**Measure**	**Minimum**	**Maximum**	**Mean**	**s.d**.
Wechsler intelligence scale—FSIQ	90.0	153.0	113.3	11.8
Johnson O'Connor vocabulary	3.0	23.0	12.8	4.5
Johnson O'Connor paper folding	4.0	54.0	27.9	13.9
Johnson O'Connor foresight	26.0	95.0	50.5	14.4
BFAS neuroticism	11.5	39.5	25.5	5.8
BFAS extraversion	22.5	49.0	35.0	4.8
BFAS openness	31.0	46.0	39.2	3.8
BFAS agreeableness	19.0	48.0	38.8	5.4
BFAS conscientiousness	23.5	45.0	34.8	5.5
Creative achievement questionnaire	1.0	96.0	18.6	19.2
HIQ engagement	3.0	10.0	8.0	1.8
HIQ implement	1.0	10.0	5.5	2.5
HIQ implement idea	1.0	10.0	7.3	2.7
HIQ difficulty	1.0	9.0	3.4	2.5
HIQ process	1.0	10.0	4.7	2.3
HIQ time spent	1.0	10.0	4.7	2.4
HIQ satisfaction	1.0	10.0	6.0	2.6
HIQ learning	1.0	10.0	5.9	2.5
HIQ experience	3.0	10.0	7.9	1.9
HIQ overall	2.0	10.0	7.0	1.7
HIQ total	4.0	8.1	6.0	1.0

FSIQ, Full Scale Intelligence Quotient; BFAS, Big Five Aspect Scale; HIQ, Hunter Imagination Questionnaire; s.d., Standard Deviation.

### Factor structure

We next sought to determine the underlying structure of the HIQ by conducting an exploratory factor analysis of the 10 questions answered at the end of the survey. Four factors were extracted, corresponding to broad domains, which are defined as “Satisfaction” (comprised of items 1, 7, 9, and 10), “Implementation” (comprised of items 2 and 3), “Learning” (comprised of item 8), and “Process” (comprised of items 4 and 5). The rotated factor matrix is presented in Table 2.

**Table 2:** **Rotated factor matrix of the Hunter Imagination Questionnaire**.

	**Factor**			
	**Satisfaction**	**Implementation**	**Learning**	**Process**
Overall	0.822			
Experience	0.703			
Engagement	0.625			
Satisfaction	0.621			
Implement 1		0.815		
Implement 2		0.811		
Learning			0.754	
Process				0.641
Difficulty				−0.450

### Reliability and validity

We next sought to determine the reliability of the HIQ by means of internal consistency of questions across factors consisting of multiple, positively related, measures. Cronbach's alpha for Satisfaction was 0.76, and for Implementation was 0.79, suggesting acceptable internal consistency of the measure, particularly across measures of Satisfaction and Implementation. Seventy-one of the original 80 participants were administered the HIQ on a second occasion, with at least 1 month of time between administrations (range 4–8 weeks). Cronbach's Alpha was 0.75 for the HIQ Total Score, suggesting good test-retest reliability for this measure. Finally, we sought to determine the correlation between the HIQ and established measures of creative achievement via the CAQ across all participants. While we found low, non-significant, correlations between the total HIQ and total CAQ, controlling for sex, (HIQ-CAQ *r* = 0.13, ns) we found significant correlations between the Implementation factor and Scientific Achievements (*r* = 0.26, *p* = 0.02), and between the Learning factor and Writing Achievements (*r* = 0.31, *p* = 0.006), suggesting concurrent validity of measures. It should be noted that these participants were over-selected for representation within the Science, Technology, Engineering, and Math disciplines; therefore, correlates between their imagination ability and Scientific Achievements might be expected to be higher than for normally selected samples.

### Brain correlates

**Figure 2:**
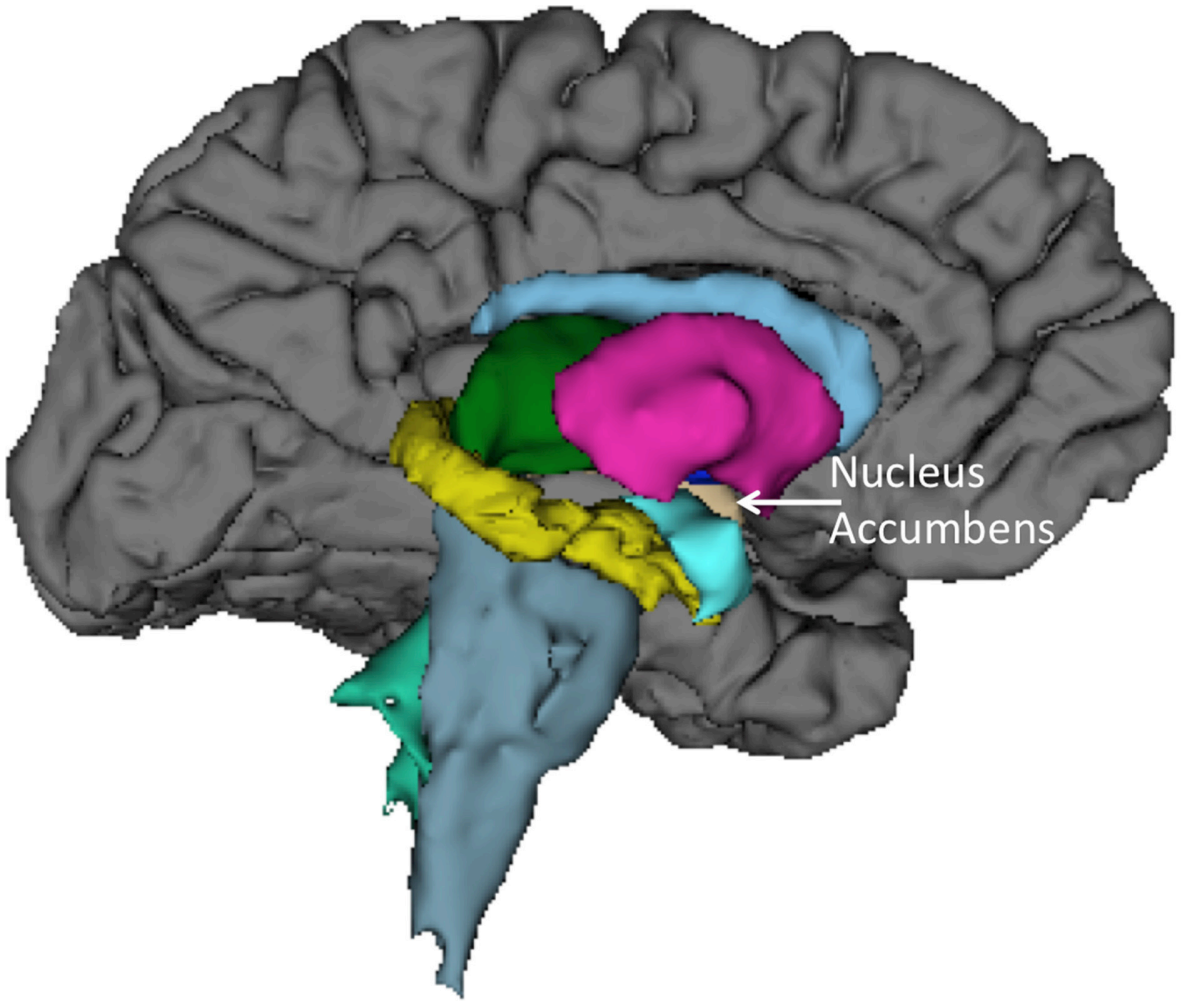
**Left medial view of average brain surface volumes (gray), and subcortical structures including the Nucleus Accumbens (light brown)**.

**Figure 3:**
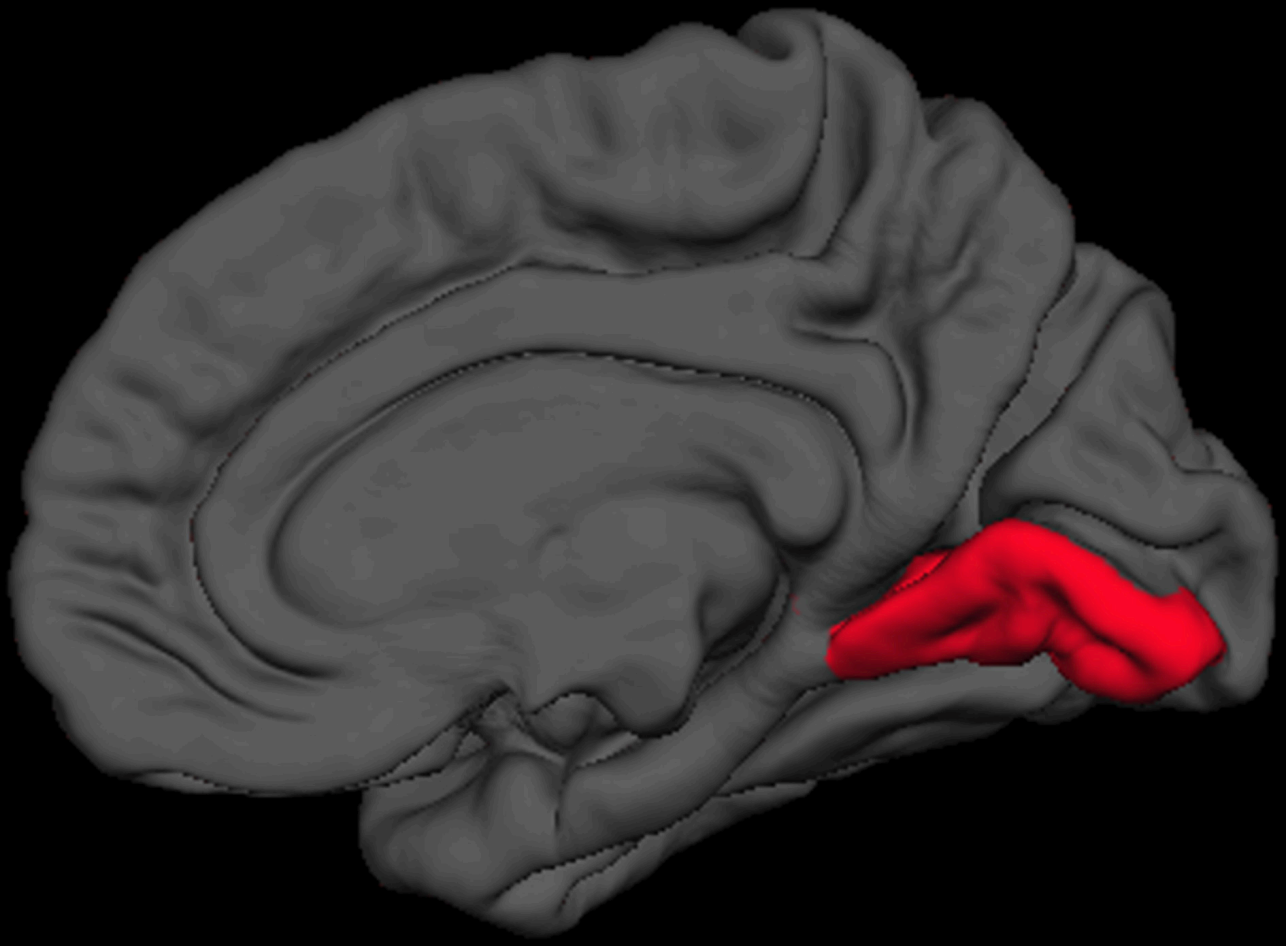
**Right medial view of average brain surface volumes (gray), showing lingual gyrus region (red)**.

Finally, we sought to determine anatomical brain correlates of the HIQ, including Factor Scores of Satisfaction, Implementation, and Learning. We regressed all volume measures, as well as subcortical volumes, against each factor controlling for Total Supratentorial volume, sex, and Full Scale Intelligence Score. The total score on the HIQ was predicted by a model that included decreased left nucleus accumbens and increased right lingual volumes (*F* = 3.3, *p* = 0.01; *r*2 = 0.18; Figure 2). A model including *decreased* volumes in the left posterior cingulate, left superior temporal gyrus, and right precuneus, and *increased* volume of left caudal middle frontal, right putamen, right rostral middle frontal, right superior frontal gyri predicted Satisfaction scores on the HIQ (*F* = 5.34, *p* < 0.001; *r*2 = 0.44). Scores on the Implementation factor were predicted by a model including *decreased* volumes of the right medial-orbital frontal gyrus, and right isthmus of the cingulate gyrus, and *increased* volumes of the left hippocampus, left lingual gyrus, and left isthmus cingulate gyrus (*F* = 4.46, *p* < 0.001; *r*2 = 0.34). Finally, the Learning score was predicted by a model that included *decreased* volumes of the left nucleus accumbens and left transverse temporal gyrus, and *increased* volumes of the right lingual gyrus and right hippocampus (*F* = 5.06, *p* < 0.001; *r*2 = 0.33) (Figure 3).

## Discussion

We found that this complex, naturalistic, measure of imagination was related to a network of brain regions previously identified to be associated with various components of this complex cognitive capacity, including the bilateral hippocampi, posterior regions of the cingulate gyrus, both medial and lateral prefrontal cortical regions, and the lingual gyrus. It is both compelling and gratifying that participants could be asked to engage in a complex task of imagination, over a period of weeks, and that their self-reported measures of satisfaction, implementation, learning, and overall experience in performing the task would be correlated with key brain regions identified as being critical to key aspects of imagination ability. The HIQ was found to be a psychometrically sound instrument, with a normal distribution of scores, good internal reliability, good test-retest reliability, and good concurrent validity with measures of creative achievement (particularly Scientific Creativity and Writing from the CAQ). As would be expected given such a complex behavioral task, the relationship between imagination and brain regions was also complex, although increased left hippocampal volume was associated with higher likelihood of implementing the imagined ideas, and increased right hippocampal volume was associated with participants' perception of increased learning about their own imagination process. This is the first study to demonstrate such brain-behavior relationships in a naturalistic setting (i.e., an online questionnaire), undertaken over a period of weeks, in normal human subjects.

Some of the complexity of the brain-behavior relationships might be explained by our previous work in creative cognition research. In our recent overview of the anatomical neuroimaging studies of creativity (Jung et al., 2013), we noted two major patterns: first, we noted a significant overlap with regions within the so-called default mode network (DMN), a brain network associated with “remembering the past, envisioning future events, and considering the thoughts and perspectives of other people” (Buckner et al., 2008); second, many of the relationships were inverse—that is lower measures of brain “integrity,” including decreased cortical volume (Jung et al., 2010b), white matter fidelity (Jung et al., 2010a), brain biochemistry (Jung et al., 2009), and even overt brain lesions (Shamay-Tsoory et al., 2011; Abraham et al., 2012), were associated with higher creative ability. Our results also conform to this general pattern; indeed, we found that nearly all *decreased* volumes that related to HIQ rankings were within DMN regions, including the posterior cingulate, precuneus, medial-orbital frontal gyrus, transverse temporal gyrus, and isthmus of the cingulate gyrus. This correspondence between decreased volumes and HIQ performance within DMN regions further supports this instrument as a measure of key aspects of imagination, including (1) remembering the past, (2) envisioning the future, and (3) considering the thoughts and perspectives of other people (Crespi et al., 2016).

We also found rather consistent associations between decreased nucleus accumbens volume and higher scores across the HIQ (i.e., Total Score and Learning factor). The nucleus accumbens is a structure linked to anticipation of incentives (i.e., reward) in humans, with lesions to this structure associated with increased impulsivity (Cardinal et al., 2001), addictive behaviors (Dalley et al., 2007), and abnormalities of appetitive and aversive behaviors (Salamone, 1994). In humans, functioning of the nucleus accumbens has been critically linked to sensation seeking and novelty seeking behaviors in non-clinical populations (Abler et al., 2006). While these results are intriguing, we anticipate that future research will help to identify the specific relationship between nucleus accumbens structure and function and imagination activity and ability. Brief mention should be made of associations between the HIQ (Total score, Implementation, and Learning factors) and increased volume of the lingual gyrus. These relationships likely reflect this structures importance to encoding and recalling complex visual material (Machielsen et al., 2000), modulating and naming visual stimuli (Howard et al., 1992; Price et al., 1994), and the analysis of the logical sequence of events (Brunet et al., 2000), all likely to be important to imagination activity and ability.

There are several limitations to the current research. The main limitation for neuroimaging studies almost always includes a note of caution given the relatively small sample, and given the complexity of brain-behavioral research questions entertained. This limitation is further highlighted by the fact that our sample included roughly twice the number of females as compared to males. We do not know why females were more likely to respond to the invitation to participate in the HIQ; however, this could have created biases in our sampling that could have affected our results. We controlled for sex throughout the analyses and there was no indication that the results did not reflect relationships across both sexes. However, future studies, with larger samples comprised of equal numbers of males and females would tend to increase the inferences that could be made. Relatedly, our sample was comprised of a young, healthy, cohort and we do not know whether our results would apply to individuals older than 35 years of age. We chose a young sample to ensure that volumetric brain changes associated with normal aging, which tend to stabilize in early adulthood (Tamnes et al., 2010), and then resume in mid adulthood would not affect our results (Ardekani et al., 2007). With regard to HIQ administration, we asked participants to limit their idea generation time to 8 min, but did not create a mechanism to check whether they took longer (or significantly shorter) to complete each session. Future studies should attempt to explicitly control and/or measure this potentially important variable. Finally, because the participants acted as their own raters, it is possible that other factors (e.g., self image, mood, etc.) could have influenced the ratings. Future studies should attempt to measure and control for such factors to determine their potential influence upon HIQ ratings.

We believe this to be the first study to relate a complex, naturalistic, measure of imagination to a network of brain regions previously associated with various facets of imagination ability. The participants' responses to the HIQ were associated with volumes across a broad network of brain regions previously associated with imagination including:

(1) Bilateral hippocampi—associated with episodic memory retrieval.
(2) Precuneus, medial-orbital frontal gyrus, posterior cingulate, and transverse temporal gyrus—overlapping significantly with the DMN—associated with “remembering the past, envisioning future events, and considering the thoughts and perspectives of other people”.
(3) Nucleus Accumbens—associated with sensation and novelty seeking behavior.
(4) Lingual gyrus—associated with recall, modulation, and analysis of complex visual material.

In conclusion, the HIQ showed good psychometric qualities, and was well tolerated by all participants. It represents a broad survey of imagination ability, obtained over days/weeks, which is more naturalistic than is customarily found in either the neurosciences or the psychological sciences. It provides a reliable, valid, method by which to assess brain-behavior relationships related to this complex cognitive construct.

## Author contributions

RJ, RF, and DH each contributed to the design of the experiment. RJ, RF each contributed to the analysis of the data. RJ and DH wrote the manuscript.

## Funding

This work was funded by a grant from the John Templeton Foundation entitled “The Neuroscience of Scientific Creativity”.

### Conflict of interest statement

The authors declare that the research was conducted in the absence of any commercial or financial relationships that could be construed as a potential conflict of interest.
